# The COVID-19 Pandemic’s Impact on Health Service Utilization Among Pregnant Women in Three Nigerian States: A Mixed Methods Study

**DOI:** 10.1007/s10995-023-03820-3

**Published:** 2023-11-17

**Authors:** Bright Orji, Emily Bryce, Bartholomew Odio, Herbert Onuoha, Elizabeth Njoku, Charity Anoke, Emmanuel Ugwa, Joseph Enne, Adetiloye Oniyire, Idris Ibrahim, Emmanuel Otolorin, Kayode Afolabi, Nnenna C. Ogbulafor, Elizabeth Oliveras

**Affiliations:** 1 Jhpiego—an Affiliate of Johns Hopkins University, Abuja, Nigeria; 2 Jhpiego—an Affiliate of Johns Hopkins University, Baltimore, MD USA; 3 Federal Medical Center, Brinin Kudu, Nigeria; 4 State Ministry of Health, Minna, Niger Nigeria; 5 Reproductive Health Division, Federal Ministry of Health, Abuja, Nigeria; 6 National Malaria Elimination Program, Federal Ministry of Health, Abuja, Nigeria

**Keywords:** COVID 19 pandemic, ANC, Nigeria, Service utilization, Lockdown, Service delivery, Change

## Abstract

**Objective:**

COVID-19 disrupted health service delivery and weakened global and national health systems. The objective of this study was to describe the changes in health service utilization in three local government areas (LGAs) in Nigeria and examine factors involved.

**Methods:**

A cross-sectional mixed-methods approach was used. A total of 315 pregnant women seen for antenatal care in 80 health facilities in three LGAs between October 1 and November 30, 2020, participated in exit interviews; 93 women participated in focus group discussions (FGDs). Descriptive analyses and a multivariable logistic analysis were conducted to examine associations between characteristics and decreased service utilization. Content analysis was used to identify the emerging themes related to health service utilization during the pandemic.

**Results:**

One quarter of women reported that they reduced or ceased health service. The biggest reported changes were in immunization (47 to 30%, p < 0.001) and a small but statistically significant decline in antenatal care (98.7 to 93.8%, p < 0.001) was observed. Qualitative findings show that lockdowns, transportation issues, increased costs and fear of contracting COVID-19 or being labeled as COVID-positive were the most common reasons for not seeking care during this period of the pandemic.

**Conclusions:**

The pandemic negatively impacted health service utilization amongst pregnant women in Nigeria. A better understanding of differences in state response could help inform future actions. The findings highlight the need for health systems to consider how to facilitate service utilization during a pandemic, such as providing safe transport or increasing outreach, and to minimize stigma for those seeking care.

**Supplementary Information:**

The online version contains supplementary material available at 10.1007/s10995-023-03820-3.

## Significance

*What is already known on this subject?* The COVID-19 pandemic has disrupted health systems worldwide and service utilization decreased as a result, including the utilization of maternal and child health services.

*What this study adds?* This study shows that there were significant declines in service utilization and provides insight into why pregnant woman did or did not alter their care-seeking for antenatal care and immunizations services. The qualitative results indicate that pregnant women responded to or interpreted State and facility-level responses differently, which resulted in increased or decreased utilization. These insights can inform future emergency responses and policies to build more resilient health systems and communities.

**Supplementary Information:**

The online version contains supplementary material available at 10.1007/s10995-023-03820-3.

## Background

The rapid spread of the COVID-19 pandemic has threatened global health and disrupted health-care delivery systems. The World Health Organization (WHO) reported that of 105 countries surveyed, 90% of countries reported some level of disruption due to the pandemic. (WHO, 2020) Lockdowns, social distancing, lack of transportation related to the pandemic, fear of contracting COVID-19, facility staff shortages and facility closures have all contributed to the disruption of health services. (Aranda et al., 2022; Kc et al., 2020; Organization, 2020; Semaan et al., 2020; Smriti et al., 2020) Previous studies have reported COVID-related disruptions in ANC services, outpatient service, vaccinations, deliveries and postnatal care in sub-Saharan Africa (SSA). (Ahmed et al., 2021; Shapira et al., 2021; Wanyana et al., 2021) A previous study in Nigeria found that 43.5% of pregnant women had at least one challenge in accessing RMNCH services since the COVID-19 outbreak. (Balogun et al., 2021). A 2020 modeling study estimated that the COVID-19 pandemic will increase monthly maternal deaths by 8.3–38.6%, from direct and indirect causes. (Roberton et al., 2020) Subsequently, a 2021 meta-analysis confirmed this trend, reporting that during the pandemic, stillbirths and maternal deaths increased significantly (pooled OR = 1.28, 95% CI 1.07–1.54 and pooled OR = 1.37, 95% CI 1.22–1.53, respectively), as compared to pre-pandemic. (Chmielewska et al., 2021).

The ability to access care during pregnancy is crucial for maternal and infant health, and is a basic human right. ANC provides a platform to identify high-risk pregnancies, prevent and manage disease (pregnancy-related or pre-existing), provide health education and promotion (including counseling on nutrition, facility-based delivery and postpartum care) as well as increasing use of maternal and child health services. (WHO, 2016) Immunization has been cited as one of the most important public health interventions, particularly for childhood mortality. (Remy et al., 2015) Other patient services are important for issues that may arise during pregnancy, such as illness or injury.

This study aimed to assess to what extent the COVID-19 pandemic disrupted health service utilization amongst pregnant women, whether the level of disruption varied by state and type of service and to examine the factors associated with changes in health care utilization.

## Methods

### Study Setting

This study was conducted in the three Nigerian states of Ebonyi, Ondo and Niger. In each State, the study took place in one local government area (LGA)—Ohaukwu (Ebonyi), Akure South (Ondo) and Bosso (Niger). In Ohaukwu and Bosso LGAs, the majority of people are rural farmers while Akure South is mostly urban dwellers engaged in various trades. For context, Table 1 outlines the COVID-19 response in each study site, from March to December 2020. The severity of the lockdown differed across the states. Mild lockdown restrictions were observed in Ondo and Niger States whereas stricter measures were observed in Ebonyi.

**Table 1:** Initial COVID-19 response by study site in 2020

	Ebonyi (Ohaukwu)	Ondo (Akure South)	Niger (Bosso)
When the first wave of the pandemic started	March, with first index case recorded on 26 April	March, with first index case recorded on 4 April	March, with first index case reported on 10 April
Date when more than 10 confirmed cases	22 May	16 June	24 June
Timing of first wave	June	June	November
Type of restrictions and severity of each	Stricter *First*—***23 March:**** Banned all public gatherings* *Second *—***28 March:**** additionally announced boundary closure (except for vehicles carrying food items, medical supplies or construction materials)* *Third*—***20 April:**** addition imposed dusk-to-down curfew (7 pm-to-7am)* *Fourth—****16* May**: *government allowed religious gathering but worshipers must be limited 50 at a service, use face mask and hand sanitizers.* * Fifth-* **June**: lockdown was relaxed, allowed use of tricycles, motor-bikes	Mild *First—****23 March:**** Banned public, religious and social gatherings for 14 days* *Second—****24 March:**** additionally announced immediate closure of all markets for 7 days* *Third—****2 April:**** closure of state borders prohibiting inter-state travels to the state*	Mild *First—****23 March:**** Announced movement restrictions from 8am to 8 pm* *Second—****27 March:**** banned intra and inter-state movement of people and vehicles (except for vehicles carrying food items, fuel, medical supplies and essential services)* *Third—****5 April:**** relaxed restriction order. Only allowing movement from 2 pm-10 pm daily*
Length of restrictions (before Nov 2020)	March—June, 2020	March—April, 2020	March—April, 2020
Did the facilities conduct outreach?	No, but CHWs were providing services	No, but CHWs were providing services	Yes, and CHWs were providing services

### Study Design and Population

This was a descriptive, cross-sectional mixed methods study. We conducted exit interviews and focus group discussions (FGDs) with pregnant women attending ANC services between October 1 and November 30, 2020 in 80 purposively selected health facilities. Some hard-to-reach facilities were excluded due to security challenges and were replaced with other accessible facilities in the same LGA.

Pregnant adolescent girls and women who were attending ANC at one of the 80 facilities and gave informed consent for participation were included in the study. Pregnant girls and women who were first time users were excluded because if this was their first-time utilizing care, there is no pre-pandemic frequency to which we can compare. For the FGDs, pregnant girls and women were purposively selected to create groups based on age (adolescent versus adult), gravidity (primi versus multi) and location (urban versus rural). Groups were separated by age and gravidity because we anticipated that this could affect participants’ experiences of care, care seeking behavior and willingness to discuss their experiences openly.

### Data Collection

A two-day training was held for the 15 data collectors on the use of the qualitative and quantitative tools followed by one day of field testing of the tools. Trained interviewers used a structured electronic survey to interview pregnant girls and women exiting ANC. The survey captured sociodemographic characteristics as well as the frequency and types of services accessed at health facilities before and during the pandemic. Data was stored real-time in a secure web-based research electronic data capture (REDCap) application.

A structured guide was used to conduct the FGDs, which was used to elicit information on factors that influenced health service utilization. The FGDs, which were moderated by a trained facilitator, lasted between 36 and 90 min (mean of 52 min) and were recorded and then transcribed. A notetaker also took notes of each discussion.

### Sample Size

We aimed to recruit a total sample of 240 women, which would allow us to measure a 20% reduction in visits of with a 95% confidence level. All project CHWs working at the selected sites were asked to contact pregnant women in need of facility-based services and invite them to the facility on the day of the interviews. All women who attended on that day were invited to participate in the survey; a total of 315 women participated in the exit interviews.

The aim of the FGDs was to triangulate and add depth to the quantitative findings, so the aim was not to obtain theoretical saturation. In total, 15 FGDs were conducted with 93 women. Each FGD had between 4 and 8 participants, depending on the availability of participants in each group. In some cases, groups were not separated by age and gravidity as planned due to low number of participants in those groups.

### Variables

The outcome of interest in this study was defined as a reduction or cessation of health services during the COVID-19 pandemic, the comparison being a report of maintaining or increasing utilization. Independent variables were age (adolescent, defined as 19 years of age or younger, or adult defined as 20 years of age or older), religion (Christian or Muslim), location (urban or rural), frequency of attendance pre-pandemic (weekly/bi-weekly versus monthly/bi-monthly) and State.

### Data Analysis

Descriptive analyses were reported in frequencies and percentages. The equality of proportions of women utilizing three separate health services before and during the pandemic were compared and the proportions and corresponding p-values were reported. Chi-squared tests and a multivariable logistic regression analysis was then used to identify factors associated with reduced or ceased utilization, controlling for potential confounders. The variance inflation factor (VIF) was estimated to assess multicollinearity, of which none was found (VIF = 1.80).

Two coders with previous qualitative experience worked under a supervisor and had a workshop during which a codebook was developed, and concepts defined based on the transcripts. They coded a few transcripts separately and compared notes. Where there are divergent views they worked with the supervisor to resolve the issue. This continued until reliability was achieved. The remaining transcripts were then coded and thematic data were extracted.

### Ethical Considerations

We received ethical approval from the Johns Hopkins School of Public Health Institutional Review Board (#00014000), and the National Health Research Ethics Committee of Nigeria (NHREC/01/01/2007-06/10/2020). Written informed consent was obtained from each respondent after explaining the details of the study. Participation in the study was voluntary and the information obtained was handled with strict confidentiality.

## Results

### Quantitative Results

There were 315 pregnant women surveyed, 15 were removed due to exclusion criteria and six were dropped due to incomplete data, resulting in a final analytical cohort of 294 women. The greatest proportion of women were from Ondo (36.0%) (Table 2). The majority of women were Christian (69.1%) and there were slightly more rural (53.7%) than urban (46.3%) dwellers. Two-thirds of participants reported utilizing health services monthly or bi-monthly prior to the COVID-19 pandemic.

**Table 2:** Sociodemographic characteristics of the pregnant girls and women (N = 294)

Variable	Number	Percentage (%)
State		
Ebonyi	99	33.7
Niger	89	30.3
Ondo	106	36.0
Age group		
Adolescent	39	13.3
Adult	255	86.7
Religion		
Christian	203	69.1
Muslim	91	30.9
Location		
Rural	158	53.7
Urban	136	46.3
Community resident		
No	18	6.1
Yes	276	93.9
Utilization frequency pre-pandemic		
Weekly/Bi-weekly	103	35.0
Monthly/Bi-monthly	191	65.0

**Fig. 1:**
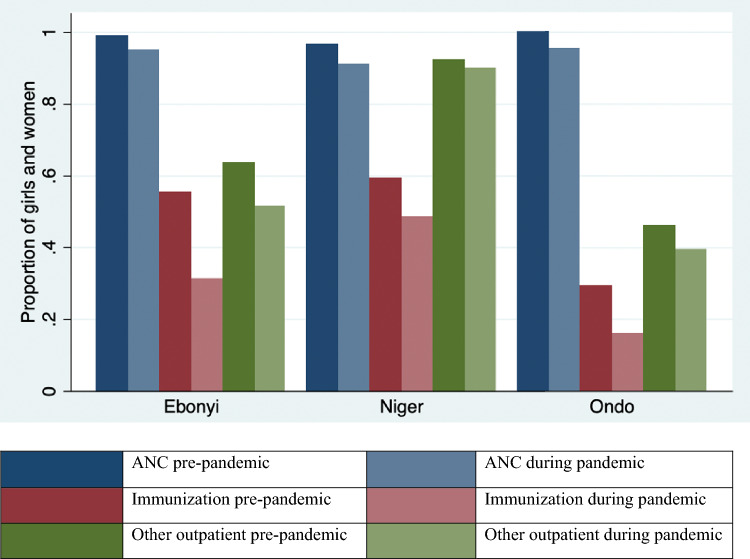
Health service utilization before and during the pandemic, by State

Reported utilization of all the measured health services decreased during this period of the COVID-19 pandemic (Table 3). ANC services witnessed a small, but significant decline from 98.7% before COVID-19 to 93.8% during COVID-19 (p < 0.05). The largest change was observed for immunization services (47.2 to 30.9%, p < 0.01). Figure 1 illustrates that the reduced utilization trend was seen in all three states, though the magnitude of the reduction varies by state.

**Table 3:** Assessment of health service utilization rates before and during the pandemic

Service	% Accessing service before the pandemic	% Accessing service during the pandemic	P-values
Antenatal care	98.7	93.8	0.0023
Immunization	47.2	30.9	< 0.0001
Other outpatient services	66.0	58.8	0.0738

When asked if their health-seeking behavior changed since the start of the pandemic, the majority (70.4%) of pregnant women responded that they have maintained the same level of utilization. Approximately 10% reported that they ceased all utilization and 11.9% reported that they reduced their utilization during the pandemic. There were 22 respondents (7.5%) that reported increased utilization. Of those that reduced or stopped utilization, 44% reported that this change was related to fear of contracting COVID-19. Table 4 presents the distribution of respondent characteristics between two groups: those women who maintained or increased utilization (N = 229) and those that reduced or ceased utilization (N = 65). There were significant differences by state in the proportion of women who reported reducing or ceasing health service utilization; compared to Ebonyi state, where 36% of women reported reducing or ceasing utilization, the odds were lower in Niger (OR = 0.52, 95% CI 0.17–1.54) and Ondo (OR = 0.43, 95% CI 0.24–0.95). Muslim women tended to reduce or cease care as compared to Christian women, but after adjusting for other covariates this was not statistically significant. Women who reported less frequent utilization pre-pandemic (monthly or bi-monthly users) were more likely to have ceased or reduced utilization during the pandemic than more frequent users (weekly or bi-weekly users), though this was not statistically significant (OR = 1.31, 95% CI 0.24–1.29). There were minor and statistically insignificant differences in utilization by place of residence and age group.

**Table 4:** Distribution of characteristics and association with health service utilization

Variable	Frequency of health service utilization during pandemic				χ^2^	χ^2^ p-value	Adjusted OR (95% CI)
	Maintained or increased		Reduced or ceased				
	N	%	N	%			
State							
Ebonyi	66	66.7	33	33.3	11.223	p < 0.05	REF
Niger	76	85.4	13	14.6			0.52 (0.17–1.54)
Ondo	87	82.1	19	17.9			0.43 (0.24–0.95)*
Religion							
Christian	150	73.9	53	26.1	6.092	p < 0.05	REF
Muslim	79	86.8	12	13.2			0.58 (0.20–2.15)
Place of residence							
Rural	123	77.9	35	22.1	0.004	0.985	REF
Urban	106	77.9	30	22.1			0.88 (0.49–1.58)
Age group							
Adolescent	29	74.4	10	25.6	0.326	0.568	REF
Adult	200	78.4	55	21.6			0.94 (0.41–2.15)
Frequency of facility use pre- pandemic							
Weekly/Bi-weekly	79	76.7	24	23.3			REF
Monthly/Bi-monthly	150	78.5	41	21.5	0.131	0.718	1.31 (0.24–1.29)

*p < 0.05; OR adjusted odds ratio

### Qualitative Results

The following six major themes emerged as factors responsible for the changes in health service utilization: (1) Fear of COVID-19; (2) COVID-19 safety measures/restrictions; (3) care-associated costs (4) Quality/efficiency of care (5) Accessibility of drugs/commodities and (6) Accessibility of health services.

#### Fear of COVID-19

The fear of having COVID-19 may have resulted in increased utilization, as women reported that individuals with symptoms often sought testing and care at health facilities immediately.

“The signs and symptoms of COVID-19 such as headache, high fever, cough is almost the same as the signs and symptoms of malaria; so when people notice these signs and symptoms, they quickly rush down to the hospital to be sure it is not COVID-19” (FGD, Ondo).

However, the fear of COVID-19 affected people differently, as other respondents reported avoiding health facilities in fear of contracting COVID-19 or testing positive at the facility.

“When corona started, pregnant women were avoiding coming to ANC because of fear of contracting the coronavirus, so the frequency of ANC attendance was reduced” (FGD, Ebonyi)”

#### COVID-19 Safety Measures/Restrictions

As expected, lockdown was reported to have reduced health service utilization because people were instructed to stay at home.

Stay at home order made us not to come to the hospital to know whether the health workers were available or not.” (FGD, Ebonyi).

Preventative measures taken by health workers may have contributed to sustained utilization during the pandemic, as women from all three states confirmed that preventive measures were enforced at the health facilities.

“When it was initially announced that everyone should stay at home and not step out or go to work. I was scared and I stayed at home. So, when they also said that we should be observing social distancing, wear face masks and wash hands, then people started coming out.” (FGD, Ondo).

#### Care-Associated Costs

In all the states, increased costs, including those for medication and face masks, were cited as a barrier to utilization since the pandemic began.

“Before corona, we were pampered each time we come for ANC, we even receive care without paying, but when corona started, we were asked to pay for everything before we are attended to.” (FGD, Ebonyi).

“Pregnant women are no longer coming in large number as before COVID-19 because of increase in hospital bill.” (FGD, Niger).

#### Quality and Efficiency of Care

Reduced waiting time and improved attitudes of health care workers and perceived quality of care were observed to have increased ANC utilization in all three states. Additionally, in all but two FDGs, staffing was reported to be adequate and that health worker availability had not changed since the start of the pandemic. However, some pregnant women in Ondo still believed that there were reduced services and interactions between health workers and client.

“There has been improvement in the service delivery. The health care workers are empathetic and check on us. They attend to patients very well.” (FGD, Ondo).

“During the COVID- 19 period they don't keep us waiting when we come for [ANC] care, they usually attend to us faster than before.” (FGD, Ebonyi).

They have stopped checking the baby’s position. Nobody tells us anymore. When they do, they only write it in the card and submit.” (FGD, Ondo).

#### Accessibility of Drugs/Commodities

Women in all three states mentioned access to routine drugs as an important factor both within and beyond the pandemic context. The reports of drug availability varied within states, where depending on the FGD, respondents reported no change in availability or limited availability or increased costs.

“They used to provide us drugs for free. sometimes we pay little money depending on the type of the drug but now everything is expensive.” (FGD, Niger).

“There have been no changes or restrictions on receiving services. They now provide more services than previously, as we are now provided with drugs for ourselves and our children.” (FGD, Ondo).

#### Accessibility of Health Services

Transportation and long distances were mentioned as challenges to accessing care during the pandemic.

“The number of pregnant women visiting the health center reduced, transportation was also a major problem because okada [motorbike taxi] was not available to bring people to the hospital. The distance to the hospital is far so it was difficult for pregnant women to trek to the health center for ANC.” (FGD, Ebonyi).

“This may make them feel like skipping their appointments because of long distance with no means of transportation.” (FGD, Ondo).

## Discussion

In our study, there were reductions in health service utilization amongst pregnant women during the COVID-19 pandemic. The largest reduction was in immunization services and there was a small, but statistically significant, reduction in ANC. Although there were reductions in utilization in all three states, the magnitude varied by state, where Ebonyi had the highest proportion of women who reported ceasing or reducing their health service utilization. The pandemic presented barriers that contributed to the decline in utilization; these included government mandated lockdowns, fears of contracting COVID-19 or being labeled COVID-19 positive, limited transportation and increased costs. However, there were aspects that mitigated these barriers and contributed to the sustained care seeking reported by the majority of our respondents, including the quality and efficiency of care and implementation of preventative measures.

The reductions in utilization in the two service areas in our study align with previous study findings. (Ahmed et al., 2021; Shapira et al., 2021; Shikuku et al., 2020; Wanyana et al., 2021) Similar to our findings, Shapira et al. reported major reduction in outpatient services and persistent but minimal reductions in ANC in Nigeria and seven other sub-Saharan Africa countries. A similar pattern of change has also been documented in ANC, postnatal care and family planning in another Nigerian study and one in Kenya. (Balogun et al., 2021; Shikuku et al., 2020) From our qualitative findings, the use of PPE, quality of interactions with health care providers and shorter waiting times resulted in women continuing to utilize services, which may be why the declines in care in our study and others were not as large as one might expect during the pandemic. Similarly, a study in Ethiopia found that women who report wearing facemasks were over twice as likely to utilize care. (Temesgen et al., 2021) However, increased costs of care, including purchasing masks, was a commonly reported negative consequence of the pandemic on health service utilization. A potential solution is provision of free PPE at all facilities; this was mentioned in certain FGDs, but not all.

The magnitude of reduction in health service utilization varied by state, with Ebonyi state having the greatest proportion of women reporting reducing or ceasing utilization during the pandemic. Several reasons could be responsible, such as the state Government’s stricter responses to the pandemic following the index case in Nigeria in March, 2020 which potentially led to differences in the COVID-19 related measures (as presented in Table 1). For instance, the severity of lockdowns, social-distancing and limited number of people gathering per time, inter and intra state travel bans, enforcement of the use of PPEs were in line with some public health measures highlighted in previous study. (Grepin et al., 2021) In the FGDs, transportation issues also came up more frequently in Ebonyi, reflecting the severity of the lockdown in this state. Lockdowns and resulting lack of transportation are commonly reported barriers to utilization, as reported by other studies. (Balogun et al., 2021; das Neves Martins Pires et al., 2021; Tadesse, 2020) Distance to a health facility and access to transportation are commonly cited barriers outside of a pandemic, so the limitations imposed by the pandemic likely exacerbated this issue. In Ondo, respondents reported that outreach by CHWs was a facilitator of service utilization. Interestingly, this was not reported in Niger, where outreach was also conducted (Table 1), though this might be due in part to the quality of the transcripts in this state, which were considerably less detailed than in others.

Some respondents reported that fear of having COVID-19 resulted in increased care seeking, as people wanted to get tested and receive care as soon as possible after symptom onset. This may have contributed to women reporting same or higher utilization, as the question asked about seeking services at health facilities broadly, which includes care-seeking for illness. However, of those that reduced or ceased utilization in our study, nearly half (44%) reported this was because of fear of contracting COVID-19. This is reflected in other studies, indicating that of those that changed their behavior, it was often to protect themselves and their families from infection. (Tadesse, 2020; Temesgen et al., 2021).

In all three states, the majority of the FGD participants reported that health worker availability had not changed since the start of the pandemic, a finding similar to another study conducted in Lagos, Nigeria. (Balogun et al., 2021) These findings are different from what has been reported in other settings, where staff shortages are cited as barriers to care. (das Neves Martins Pires et al., 2021; Organization, 2020; Semaan et al., 2020) The availability of health workers in our study may have contributed to the small decline of five percentage points in ANC utilization seen during the pandemic. However, specifically in Ondo State, some of pregnant women complained of reduced client-provider interactions and feedback which resulted in limited access to services within ANC clinic such as health and counselling sessions and abdominal examinations as routinely conducted before the pandemic, which violates the basic ANC guidelines as recommended by WHO. (WHO, 2016) This suggests that within a state, there may be variation in perceptions or attitudes by facility, or by provider.

### Limitations of the Study

The study was limited to small number of pregnant women in three LGAs (Ohaukwu, Bosso and Akure) that represent three zones in Nigeria (South-East, South-West and North-Central). This may limit the generalizability of the results, even within Nigeria. As mentioned earlier, the quality and richness of the qualitative interviews varied between states, which limited our ability to make inter-state comparisons within the qualitative data and limited triangulation with the quantitative data. Another limitation is that women were asked to report health service utilization pre-pandemic, which introduces the possibility for recall bias. A final limitation is that the sample was taken from women seeking antenatal care, and thus may not be generalizable to other populations.

## Conclusion

Concerns about pregnancy-related services during COVID-19 and the resulting struggling status of the health care systems has awakened nations, including the Government of Nigeria on the need to assess the extent of change in health service utilization and identify factors responsible for the changes during COVID-19 pandemic. Health service utilization differed significantly by State, paralleling differences in both levels of transmission and the State-level response to the pandemic. Despite all health service areas seeing a reduction in utilization, mediators such as use of PPE, social distancing and quality of care by providers were reported to be facilitators for utilization during the pandemic. A better understanding of differences in the pandemic severities and state response could inform future actions in Nigeria, particularly in how to better facilitate service utilization and address public fears and stigma. Future research and programmatic efforts should focus on improving outreach campaigns, strengthening information-sharing channels (including telehealth), and informing context-specific approaches for preventative measures against the pandemic.

### Supplementary Information

Below is the link to the electronic supplementary material.

Supplementary file2 (XLSX 70 KB)

Supplementary file1 (DTA 253 KB)

## Data Availability

The datasets used and/or analyzed during the current study are included in this published article as an Excel spread sheet/Stata (and its supplementary information files). In addition, it is available from the corresponding author on reasonable request.

## Abbreviations

ANC: Antenatal Care
CHC: Community Health Centre
CHW: Community Health Worker
FGD: Focus Group Discussions
HC: Health Centre
LGA: Local Government Area
OPD: Outpatient Department
OR: Adjusted Odds Ratio
PHC: Primary Health Center
PPE: Personal Protective Equipment
SSA: Sub-Saharan Africa
WHO: World Health Organization

## Code Availability

(Software application or custom code)—Not applicable.
